# O.2.3-10 Insights into the implementation of workplace health promotion interventions among education professionals using a participatory approach

**DOI:** 10.1093/eurpub/ckad133.136

**Published:** 2023-09-11

**Authors:** Liesbeth Groenesteijn, Anass Arrogi, Piet Verstegen, Petra Biemans, Jasmijn Holla

**Affiliations:** Faculty of Health, Sports and Social Work, Inholland University of Applied Sciences, Haarlem, The Netherlands; Centre of Expertise Prevention in Health and Wellbeing, Inholland University of Applied Sciences, Haarlem, The Netherlands; Faculty of Health, Sports and Social Work, Inholland University of Applied Sciences, Haarlem, The Netherlands; Centre of Expertise Prevention in Health and Wellbeing, Inholland University of Applied Sciences, Haarlem, The Netherlands; Faculty of Business, Finance and Law, Business Research Centre, Inholland University of Applied Sciences, Amsterdam, %he Netherlands; liesbeth.groenesteijn@inholland.nl; Faculty of Business, Finance and Law, Business Research Centre, Inholland University of Applied Sciences, Amsterdam, %he Netherlands; liesbeth.groenesteijn@inholland.nl; Faculty of Health, Sports and Social Work, Inholland University of Applied Sciences, Haarlem, The Netherlands; Centre of Expertise Prevention in Health and Wellbeing, Inholland University of Applied Sciences, Haarlem, The Netherlands

## Abstract

**Purpose:**

The main purpose of this explorative study was to examine implementation strategies of workplace health promoting interventions for employees of a large education institution. The corresponding objectives were to identify facilitators and barriers for implementation, to define needs and wishes of employees regarding health promotion and to develop suitable interventions and implementation strategies. The health promoting interventions and strategies were gathered using an innovative participatory approach.

**Methods:**

We used a collaborative participatory action research (PAR) approach among delegates of teachers, managers and HR professionals with three groups (5-6 delegates) representing three divisions of the organization. In four 3-hour co-creation sessions each group of delegates discussed health promoting intervention strategies including implementation facilitators and barriers, wishes and needs of their colleagues and implementation strategies. The sessions were facilitated and guided by researchers and data were collected in written anonymous reports. The researchers thematically analyzed the data and presented the collected data in a final session. In the final session, representatives of the three delegate groups shared the data and discussed common suitable health promoting intervention strategies.

**Results:**

The co-creation sessions resulted in a variation of facilitators and barriers on multiple levels of implementation. We identified common facilitators at the individual (e.g. individualized approach), team (e.g. social inclusiveness) and organizational level (e.g. wide health promotion initiatives). Conversely, common barriers were identified at the individual (e.g. lack of time), team (e.g. lack of manager support) and organizational level (e.g. hampered digital accessibility).

Main topics derived from the wishes and needs include a facilitating (healthy) work environment (e.g. healthy food choices) and social meeting options (e.g. group physical activities). These wishes and needs were accompanied with facilitating prerequisites (e.g. flexibility and autonomy).

Lastly, the co-creation sessions provided suitable intervention avenues including an in-house health promotion center backed by an accessible digital environment (intranet).

**Conclusions:**

This participatory approach provided tailored workplace health promoting interventions and offered implementation strategies targeting education professionals. These implementation strategies will be pilot-tested in phases across multiple divisions within the education institution.

**Support/Funding Source:**

The study was internally funded by the Inholland University of Applied Sciences.

